# The activity of combinations of currently used antigiardial drugs against *Giardia duodenalis*

**DOI:** 10.1093/trstmh/traf101

**Published:** 2025-09-18

**Authors:** Keely Fayd'Herbe, Chun Kit Lam, Christopher J S Hart, Tina S Skinner-Adams

**Affiliations:** Institute for Biomedicine and Glycomics, Griffith University, Nathan, Queensland, 4111, Australia; School of Environment and Science, Griffith University, Nathan, Queensland, 4111, Australia; Institute for Biomedicine and Glycomics, Griffith University, Nathan, Queensland, 4111, Australia; School of Environment and Science, Griffith University, Nathan, Queensland, 4111, Australia; Institute for Biomedicine and Glycomics, Griffith University, Nathan, Queensland, 4111, Australia; School of Environment and Science, Griffith University, Nathan, Queensland, 4111, Australia; Institute for Biomedicine and Glycomics, Griffith University, Nathan, Queensland, 4111, Australia; School of Environment and Science, Griffith University, Nathan, Queensland, 4111, Australia

**Keywords:** drug combinations, Giardia, treatment-refractory giardiasis

## Abstract

**Background:**

Drug-combination therapies are needed to combat treatment refractory giardiasis. However, data describing the activity of combinations of currently used drugs against *Giardia duodenalis* are unavailable.

**Methods:**

The in vitro activity of combinations of currently used antigiardial drugs were investigated against *G. duodenalis*.

**Results:**

Combinations of metronidazole with albendazole or quinacrine, and quinacrine with nitazoxanide, behaved additively, whereas combinations of metronidazole with nitazoxanide, albendazole or quinacrine behaved synergistically. Combinations of albendazole with nitazoxanide behaved antagonistically.

**Conclusions:**

While combinations of metronidazole with nitazoxanide, or albendazole with quinacrine, may be effective treatments of giardiasis, combinations of albendazole with nitazoxanide are likely to result in negative pharmacodynamic interactions.

## Introduction

Giardiasis, caused by the flagellate protozoan parasite *Giardia duodenalis*, is a common but neglected intestinal illness that results in global morbidity. As there are no vaccines for giardiasis, control relies on sanitation, education and chemotherapies. However, current chemotherapeutic options are failing, resulting in increasing reports of treatment refractory disease. Indeed, up to 50% of infections have been reported to be treatment refractory.^1^ To combat treatment refractory giardiasis, there is an urgent need for new drugs and drug combinations that improve treatment outcomes and prevent the spread of drug-resistant parasites.

While drug-combination strategies have demonstrated their worth in controlling other parasites, they are rarely used to treat giardiasis. There are also limited data available that describe the activity of drug combinations against *G. duodenalis*.^1,2^ In vivo activity assessments are essentially limited to case studies or trials involving small cohorts,^2^ and despite providing important pharmacodynamic interaction information that can guide the selection of drug-combination partners, only one combination of currently used frontline antigiardial drugs, metronidazole with quinacrine, has been examined in vitro.^3^ To improve this position, the aim of the current study was to investigate the in vitro activity of combinations of currently used antigiardial drugs against *G. duodenalis*. Combinations assessed included the 5-nitroimidazole, metronidazole, the benzimidazole, albendazole, the thiazolide, nitazoxanide, and the acridine derivative, quinacrine.

## Materials and methods

The activity of drugs whether alone or in combination was assessed against BRIS/87/HEPU/713 *G. duodenalis* parasites in 96-well micro titre plates (2% O_2_, 5% CO_2_ in N_2_) using automated microscopy.^4^ Media, parasite and dimethyl sulfoxide (DMSO)-vehicle controls (0.4%) were included on each plate and all tests were performed in biological triplicate with growth assessed after 48 h. Drug combinations were assessed as previously described,^5^ by titrating an eight-point concentration range of each drug against seven fractional inhibitory concentrations (FICs; 0–1; where an FIC=1 was equivalent to each drug's IC_50_ as determined using non-linear regression analysis [GraphPad Prism, Dotmatics, San Diego, California, USA]) of the remaining drugs. Data were visualised by generating isobolograms, plots of drug combinations that resulted in 50% growth inhibition. Isoboles were fitted to these data and interaction (I) values calculated as previously described.^5^ Using this analysis, positive I values indicate synergy, negative I values indicate antagonism and I=0 indicates additivity. The significance of I from 0 was assessed using Student's t test.^5^

## Results

**Figure 1.**
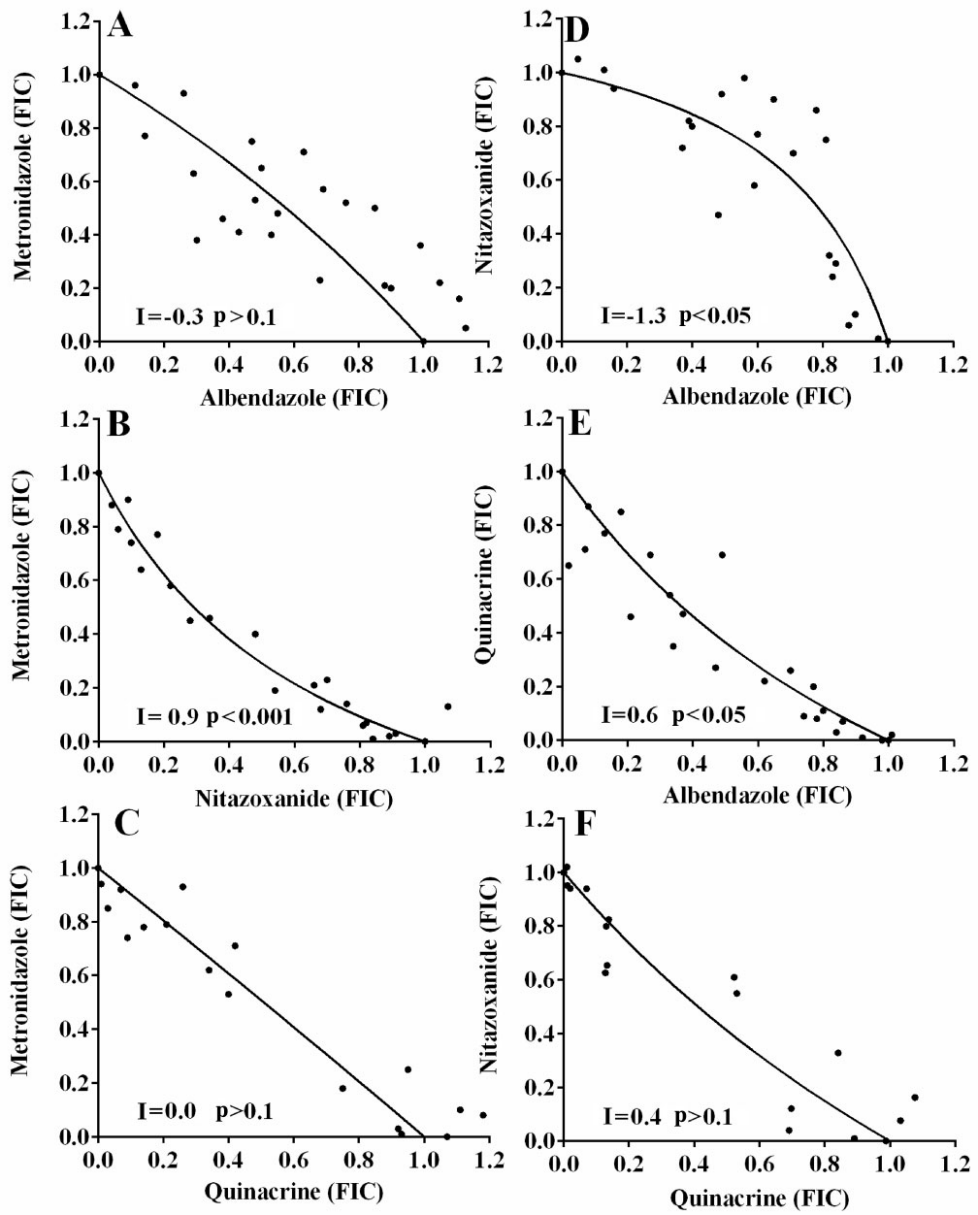
Isobolograms illustrating the interactions of antigiardial drug combinations against *G. duodenalis* parasites *in vitro*. The *in vitro* interaction of metronidazole in combination with albendazole (A), nitazoxanide (B), quinacrine (C), albendazole in combination with nitazoxanide (D) and quinacrine (E) and quinacrine in combination with nitazoxanide (F) were assessed by performing 48 h checkerboard growth inhibition studies against BRIS/87/HEPU/713 *G. duodenalis* parasites and generating isobolograms, plots of combinations of compounds that resulted in 50% growth inhibition.^5^ Combinations of compounds that resulted in 50% growth inhibition are presented as fractional inhibitory concentrations, where a FIC=1 represents the concentration of each compound required to inhibit *G. duodenalis* growth by 50%. Interaction (I) values and the significance of these values from zero was assessed as previously described.^5^

The 48 h growth inhibition activities of metronidazole (IC_50_ 4.2±0.7 µM), albendazole (IC_50_ 0.8±0.1 µM), quinacrine (IC_50_ 1.0±0.2 µM) and nitazoxanide (IC_50_ 0.6±0.3 µM) were within previously reported ranges.^2,4^ When assessed in combination against *G. duodenalis* parasites in vitro, metronidazole behaved additively with albendazole (I=−0.3; p>0.1; Figure 1A) or quinacrine (I=0.0; p>0.1; Figure 1C), but synergistically with nitazoxanide (I=0.9; p<0.001; Figure 1B). Albendazole behaved antagonistically with nitazoxanide (I=−1.3 p<0.05; Figure 1D) and synergistically with quinacrine (I=0.6 p<0.05; Figure 1E). Meanwhile, combinations of quinacrine with nitazoxanide behaved additively (I=0.4 p>0.1; Figure 1F).

## Discussion

The data generated from this work suggest that while most combinations of currently used antigiardial drugs behave additively or with some synergy in vitro and hence may be useful in vivo, caution should be advised when using albendazole with nitazoxanide. This drug combination behaved antagonistically against parasites in vitro (Figure 1D) and hence may not be the best combination to use against treatment refractory parasites. While further studies are required to determine whether this combination is antagonistic in vivo, a 2013 randomised controlled trial investigating the activity of nitazoxanide and albendazole in children, against *Trichuris trichiura*, demonstrated this combination to have poor activity against *G. duodenalis*.^6^

In contrast to the unfavourable activity of nitazoxanide and albendazole, the observation that albendazole behaved synergistically with quinacrine bodes well for the use of this combination in vivo. Noting that while there has been only one report of quinacrine and albendazole therapy being used in a treatment refractory setting and that there are some concerns regarding the safety of quinacrine, this study reported 100% efficacy (7/7 cases).^1^ Data generated during the current study also support combatting treatment refractory giardiasis with metronidazole and nitazoxanide combination therapies. While the synergistic activity of these drugs was not anticipated given the ability of nitazoxanide to inhibit pyruvate ferredoxin oxidoreductase, and the importance of this enzyme in reducing metronidazole to its reactive species,^2^ these data highlight the potential of this combination. They also demonstrate the importance of experimentally examining the direct activity of compound combinations, given that the overall impact of these therapies may be the result of a complex interplay of interactions that are rarely completely understood.

## Conclusions

The current study has identified synergistic drug combinations that may help combat treatment refractory *G. duodenalis* infections and hence should be prioritised for further in vivo safety and efficacy investigations. Evidence demonstrating that albendazole and nitazoxanide behave antagonistically in vitro may explain why this combination has demonstrated poor activity in the clinic and suggest that this drug combination should not be used in treatment refractory cases.

## Data Availability

All raw data underlying this article will be shared on reasonable request to the corresponding author.

## References

[1] Morch K, Hanevik K. Giardiasis treatment: an update with a focus on refractory disease. Curr Opin Infect Dis. 2020;33(5):355–64. 32773501 10.1097/QCO.0000000000000668

[2] Riches A, Hart CJS, Trenholme KR et al. Anti-giardia drug discovery: current status and gut feelings. J Med Chem. 2020;63(22):13330–54. 32869995 10.1021/acs.jmedchem.0c00910

[3] Gillin FD, Diamond LS. Inhibition of clonal growth of *Giardia lamblia* and *Entamoeba histolytica* by metronidazole, quinacrine, and other antimicrobial agents. J Antimicrob Chemother. 1981;8(4):305–16. 6271724 10.1093/jac/8.4.305

[4] Hart CJ, Munro T, Andrews KT et al. A novel in vitro image-based assay identifies new drug leads for giardiasis. Int J Parasitol Drugs Drug Resist. 2017;7(1):83–9. 28171818 10.1016/j.ijpddr.2017.01.005 PMC5295624

[5] Skinner-Adams TS, Fisher GM, Riches AG et al. Cyclization-blocked proguanil as a strategy to improve the antimalarial activity of atovaquone. Commun Biol. 2019;2(1):166. 31069275 10.1038/s42003-019-0397-3 PMC6499835

[6] Speich B, Marti H, Ame SM et al. Prevalence of intestinal protozoa infection among school-aged children on Pemba Island, Tanzania, and effect of single-dose albendazole, nitazoxanide and albendazole-nitazoxanide. Parasit Vectors. 2013;6(1):3. 23289920 10.1186/1756-3305-6-3 PMC3558385

